# The Hospital Nursing Supplement

**Published:** 1896-11-28

**Authors:** 


### "The Hospital" Nursing Mirror.

Being the Extra Nursing Supplement of "The Hospital."

[Contributions for this Supplement should be addressed to the Editor, The Hospital, 28 & 29, Southampton Street, Strand, London, W.C., and should have the word "Nursing" plainly written in left-hand top corner of the envelope.]

### News from the Nursing World.

### VICTORIA HOSPITAL FOR CHILDREN, CHELSEA.

Captain Blount, R.N., secretary to the Victoria Hospital for Children, Chelsea, is famous for the success of his efforts to obtain help for this institution, and all previous efforts bid fair to be outdone by the schemes now planning for next June in commemoration of the sixtieth year of the accession of the Queen. A grand fête and fancy fair will be held in the Botanical Gardens from the 21st to the 25th of that month in aid of the funds of the hospital. The cause of suffering children always appeals to popular sympathy, and the fact that this is the only hospital in London bearing Her Majesty's name gives appropriateness to this special effort. The Prince and Princess of Wales have consented to become patrons of the undertaking, and amongst the ladies who are to hold stalls are the Duchess of Sutherland, Lilian Duchess of Marlborough, Countess Cadogan, Countess of Aylesford, Viscountess Chelsea, and Lady Harris, &c., &c. The dramatic profession, headed by Miss Ellen Terry, as usual when there is any philanthropic scheme on foot, is well to the fore in promises of help, and amongst the patrons is Sir Henry Irving. There will be old-fashioned May-pole dances by members of the Children's Salon; Mr. Maskelyne will exhibit "A Mahatma in Pickle"; and the illuminations in the evening, with a water carnival on the lake, sound most attractive. Captain Blount has gathered together a good list of patrons, as well as a long stewards' list, to which, however, he will be very willing to add. Communications should be addressed to him at the hospital.

### ROYAL HOSPITAL, RICHMOND.

The Royal Hospital, Richmond, has interesting associations for those who know something of its history. The oldest part of the building, in which is the present entrance and a quaint, old-fashioned hall and staircase, was once the home of the poet Thompson, who is said to have written "The Seasons" in the room which is now the matron's sitting-room. Later, Lord and Lady Shaftesbury lived in the house, which was afterwards added to for its present purposes, and now contains three wards—men's, women's, and children's. The latter, opened by and named after H.R.H. the Duchess of York in the spring of this year, is a charming nursery for the small invalids who inhabit its dainty cots. The pale green tiled and distempered walls have a pleasant, fresh look, and the floor of polished oak is all that a floor should be. The large, low windows, with a glass door opening on the garden at one end, give the children the amusement of watching anything that may be going on outside from their beds. A cheerful day-room, furnished with a low children's table and miniature armchairs, leads out of the ward. It seems odd that, in such a place as Richmond, no rich friends of the hospital have undertaken to keep this and the other wards supplied with flowers. Flowers there are in almost every hospital ward nowadays, but they are usually the gift of "patients' friends," or are purchased by the nurses It is a little thing for wealthy folk to give from their abundance, but the pleasure thereby bestowed is great.

### ALEXANDRA HOSPITAL FOR CHILDREN, BRIGHTON.

The new wing just added to the Royal Hospital for Sick Children, Dyke Road, Brighton, formally opened by the chairman of the committee, Councillor Dewe, last week, provides sleeping accommodation for twelve nurses, we are sorry to note, in cubicle form instead of in separate bed-rooms. The building is at the back of the hospital, with which it communicates by a covered way; and the money, both for the erection and for fittings, has been so well subscribed that the chairman was able to announce that it was opened without any shadow of debt upon it. Mr. and Mrs. Payne presented a most welcome gift in the shape of fourteen armchairs and a clock, a substantial help towards the furnishing.

### POOR LAW OFFICERS' SUPERANNUATION ACT.

By invitation of Miss Vincent, of the St. Marylebone Infirmary, a conference of Poor Law Guardians, infirmary matrons, and others interested in the effect which the new Superannuation Act will have on nurses and other women who work under the Poor Law, was held at 12, Buckingham Street, on Tuesday afternoon. There was a good attendance, amongst those present being Miss Vincent (Marylebone), Miss Gibson (Birmingham), Miss de Pledge (Chelsea), Miss Elma Smith (Central London Sick Asylum), Miss Moir (St. Pancras), Miss Wilson (Workhouse Infirmary Nursing Association), Miss Arnit, Miss Ambler Jones, Mr. Robinson, Dr. Savill, Dr. Humphreys, and Mr. Bousfield. Mr. Robinson took the chair. The rough draft of a petition to be presented to Members of Parliament and the Local Government Board, embodying the views of the conference, was discussed and approved of. The chief points brought forward were the injustice of the present compulsory nature of the Act as regards nurses and all female officers and servants, and the need for lowering the limit of age fixed for their superannuation. It was unanimously decided that the petition should be presented in the interests of all female officers under the Poor Law, and not of trained nurses alone. A committee was appointed to finally revise the petition, and a meeting for that purpose was fixed for Wednesday next, the result of which we hope to publish in our next issue.

### MIDWIVES' REGISTRATION.

A paper by Miss Charlotte Ellis on the Midwives' Registration Bill was read at a recent meeting of the Midland Union of Women's Liberal Association, held at Leicester. The resolution, "That this conference earnestly hopes, in the interests of working women (especially in the rural districts), that the Midwives' Registration Bill may, with suitable alterations, be passed into law," was finally passed without dissent.

### NURSING BY NUNS.

We have many times urged that if nuns wish to nurse they should be trained as nurses. This common-sense view is also now urged by *Erin*, a publication edited by Mrs. Ernest Hart, in which it is stated that the nuns themselves are beginning to see the need for a higher standard of nursing, and one more approaching to that of the hospital-trained nurse. Already some of them have undergone a term of training. The sisters belonging to the "Little Company of Mary" undertake all kinds of nursing, and are trained at the Limerick Hospital, and at the convent at Hyson Green, at Nottingham, and also in Rome and Florence. The Bon Secours sisters are also trained as nurses. As Mrs. Ernest Hart very properly says: "In a country where the nuns of Foxford, Ballaghderin, and other convents have shown that, for the cause of the poor and to bring them employment, they can master the difficulties of manufacture and the intricacies of commerce, surely all nuns will be ready to practically learn hospital nursing so as to fulfil better the holy duty of caring for the sick."

### A WOMEN'S HOSPITAL.

A well-attended drawing-room meeting in aid of the Clapham Maternity Hospital, the only one of its kind managed and worked entirely by women, was held, by permission of Mrs. Charles Meyerstein, at 51, Finchley Road, on November 13th. The special object put before the audience by the speakers, who included Mrs. Fawcett, Dr. Helen Webb, Dr. Annie McCall, and Miss Ritchie (the hon. secretary and treasurer of the hospital), was the urgent need of raising a fund for the erection of a proper hospital building, the present accommodation being quite inadequate. Besides enabling women to be attended either in the hospital or at their own homes by doctors of their own sex, the hospital affords means for lady medical students to take their training under fully-qualified women teachers, without the presence of male students; and it is certainly time that the useful work thus accomplished should have for headquarters a building more in keeping with modern times than the adapted houses in Jeffrey's Road, which at present constitute the Clapham Maternity Hospital.

### BIRMINGHAM GUARDIANS AND THE SUPERANNUATION ACT.

The Birmingham Board of Guardians have been making themselves somewhat ridiculous by gravely discussing whether or no "probationer nurses, domestic servants, charwomen, and scrubbers" are to be defined as "permanent officers," and so to be considered as coming under the provisions of the new Superannuation Act. The *Birmingham Daily Post* states that "after some discussion" a resolution was passed at a recent meeting decreeing that probationer nurses "should be included with the other officers," while it was further decided to exclude domestic servants from "the benefits of the statute." All this appears rather a waste of time on the part of the Guardians, seeing that they have no power at all to decide who shall come under the Act and who shall not, and have only to apply to the Local Government Board for an explanation of the meaning of the Act, should they not understand it, to the terms of which they are bound to adhere while it remains law.

### DISTRICT NURSING AT BRIGHTON.

The new District Nursing Association, just started at 5, Marlborough Place, Brighton, largely through the efforts of Mrs. Uhthoff, was formally opened on the 11th inst. by the Countess of Chichester. The Mayor presided over a well-attended meeting, at which the Bishop of Chichester and the Vicar of Brighton were present. The association is affiliated with the Queen's Jubilee Institute. Several interesting speeches were made. Mrs. Cheadle read a paper on the subject of district nursing generally; Miss Gray, of the Metropolitan Nursing Association, explained the system followed in the training of "Queen's Nurses," and a good deal of very encouraging interest was evinced on all sides. We wish the new institution every success.

### BERI-BERI.

A correspondent writes asking for information as to the disease called Beri-beri, which has lately occurred in the Richmond Asylum, Dublin, where it has attacked upwards of a hundred persons, six of whom have been nurses. It is a disease which is widely spread in many parts of India, China, and Japan. It is probably due to some micro-organism. It shows itself by fever anæmia, and general anasarca, and by signs of multiple neuritis, such as numbness, loss of tendon reflexes, and muscular atrophy. Sometimes it occurs in epidemic form, the prominent symptoms being dropsy, shortness of breath, and sensory disturbances, with paralysis. The mortality varies greatly in different epidemics. It is probably not directly infectious, but those who in their dealings with it are exposed to the same influences as those under which it has attacked their patients, are prone to suffer along with them, as is shown in the Dublin cases.

### SHORT ITEMS.

The Committee of the Redruth District Nursing Association have decided to raise the salary of Nurse Brindley by £5 per annum, in "recognition of her valuable services."—The Aberdeen District Nursing Association has received a donation of £500 from the trustees of the Chalmers Fund. Miss Armstrong, the superintendent, was one of those recently appointed to receive the silver badge of the Queen Victoria's Jubilee Institute.—The Mayoress of Newcastle has been successful in raising the full amount needed to defray the cost of furnishing the houses now being used as temporary accommodation for the Royal Infirmary nurses.—At the recent annual meeting of the Bridport General Sick Nursing and Lending Society, universal satisfaction was expressed with Nurse Bradley's work. The committee reported that she had proved herself skilful and attentive, and a careful and kind friend to all her patients.—The Executive Committee of the Worcester Infirmary have accepted a tender for £4,133 for the new nurses' home.—The Prince of Wales has sent thirty pheasant to St. Saviour's Hospital, Osnaburgh Street.—A successful ball in connection with the Singer Cycling and Football Clubs, of which the proceeds were given to the Coventry District Nursing Association, was held early this month at Coventry. Lord and Lady Warwick were present.—The "Old Paris" Bazaar, just held at Sunderland in aid of the Monkwearmouth and Southwick Hospital, resulted in a net profit of £1,150.—The Russian National Health Society is taking steps to celebrate early next month the centenary of vaccination, and under its auspices an exhibition is in preparation. To this exhibition the Queen has presented an engraved portrait of Lady Mary Wortley Montague.—A district nurse has begun work at Newbridge, Kildare, in connection with the St. Lawrence Home at Dublin.

### Hygiene: For Nurses.

By John Glaister, M.D., F.F.P.S.G., D.P.H.Camb., Professor of Forensic Medicine and Public Health, St. Mungo's College, Glasgow, &c.

### XXXIV.—CHARACTERISTICS OF INFECTIVE MATERIALS OF ZYMOTIC DISEASES—INCUBATION—INVASION—INFECTIVITY—INFECTIOUS DISEASES IN RELATION TO SCHOOLS.

As we have already shown, the infective material of zymotic diseases consists of the microbe, or its spores, or both. Each infectious disease has what is termed its striking distance, *i.e.*, the area from the infected person within which the infective material will operate prejudicially on a predisposed person. But in this respect these diseases vary. For example, one has a short striking distance, another a long, and a third an intermediate. Typhus fever is illustrative of the first class. The zone of infection is confined to the immediate person of the sufferer. Small-pox is an example of the second class. It seems well-established that a small-pox hospital situated at too close proximity to populated houses is a menace to the occupants of the latter. If propagation by other means be eliminated, it would appear that the spread of infection in the neighbourhood can only be accounted for by air-borne infection.

The factor which more than all others determines this area of infectivity, is the mode or channel by which the infective substance leaves the body. In typhus it is eliminated by the mucous discharges, and by the skin, and in a form readily oxidised by the atmosphere. In erysipelas, scarlet fever, measles, and some others, it is thrown off in the exfoliated scurf-skin during convalescence, but in the febrile stage by the breath and mucous discharges. In diphtheria it does not spread far from the body of the patient, being discharged in the moist mucous throat discharges, and is consequently held in retention. This is still more marked in enteric fever and cholera; hence the non-contagious character of these diseases is well-established both in theory and in experience; when, however, these discharges become dry they are in a position to be dangerous. Indeed, it may be concluded as a general principle that where the infective material is eliminated in a dry medium, its infectivity is more pronounced, because being more easily air-borne, than where it is thrown off in a moist or distinctly wet medium; and, in like manner, contagiousness is increased.

All infective diseases pass through three stages, which have been called, respectively, incubation, invasion, and infectivity. These are, however, but arbitrary terms, and by no means indicate sequential conditions, since in certain diseases infectivity is consentaneous with incubation and invasion. The duration of these periods differs in different diseases. Cholera probably has the shortest incubation period, and rabies one of the longest. Infectivity may be said to be existent in any given case from the time the microbe multiplies in the body until it has been expelled.

Incubation may be defined as that period which comes between the growth of the specific microbe in the body and the production of fever, the result of its growth and multiplication within the body. It may be divided into two stages: first, the latent stage, during which the presence of the microbe is not marked; and, second, the invasive stage, during which the harmful effects of the micro-organism become apparent. The former has to do with the growth of the microbe itself, the latter with the effects of its growth upon the health of the patient. These, however, are only useful academic terms. Whatever view we take of incubation—in other words, of the "hatching" or "brewing" stage, as it has been called—experience shows that its duration varies in different diseases. Some have short, some long, and others intermediate incubation periods.

Short Incubation Periods (One to six days).

| | |
|---|---|
| Anthrax (in man) | 1 to $1\frac{1}{2}$ days |
| Cholera | 1 to 3 ,, |
| Diphtheria | 2 to 5 ,, |
| Erysipelas | 1 to 4 ,, |
| Glanders (in man) | 1 to 4 ,, |
| Influenza | 1 to 6 ,, |
| Foot and mouth disease | 3 to 5 ,, |
| Puerperal fever | 3 to 5 ,, |
| Scarlet fever | 2 to 4 ,, |
| Relapsing fever | about 5 ,, |

Long Incubation Periods (Seven to twenty-one days).

| | |
|---|---|
| Whooping-cough | 7 to 14 days |
| Typhus fever | 7 to 14 ,, |
| Small-pox | 10 to 15 ,, |
| Chicken-pox | 10 to 15 ,, |
| Rabies (in animals) | 6 to 24 ,, |
| Enteric fever | 12 to 20 ,, |
| Measles | 8 to 12 ,, |
| German measles | 10 to 14 ,, |
| Mumps | 14 to 21 ,, |
| Yellow fever | 2 to 10 ,, |

Variable Incubation Periods.

| | |
|---|---|
| Malaria | 6 to 20 days, or longer. |
| Rabies (man) | 3 to 70 ,, ,, |
| Tuberculosis | very variable, usually long. |
| Tetanus | ,, ,, within 10 days. |

Invasion is the period between the intervention of the febrile symptoms and the appearance of the rash—where a skin eruption is a usual phenomenon. In scarlet fever, it is from one to two days; in small-pox and typhus, from two to three days; and in measles, from three to five days.

Infectivity is that period during which the infected person discharges from his body infective material which is capable, under suitable conditions, of causing the same disease in others. Its duration is, to some extent, limited by the intensity of the attack, and the time which elapses before the complete restoration to health of the person attacked. In olden times, it was held to last for forty days From this our word quarantine is derived (Ital.: *quarantana*—a space of forty days). This was a workable and fairly safe general conclusion, as, with but few exceptions, forty days would cover the infective period of the known infectious diseases. But the period of infectivity of any given case should not be regulated so much by a fixed time-limit, as by the time when the body has got rid of the débris which usually carries the infective material. This is especially true of the eruptive infectious diseases, and of diphtheria.

The Infective Period may be taken to last in

Cholera—during the whole period of discharges and for a week thereafter.

Small-pox—during the whole illness till after the last particle of the last crust has fallen from the body, and for ten days thereafter.

Chicken-pox—the same.

Scarlet fever—during the whole illness until last particle of exfoliated skin has been shed—six to eight weeks.

Measles—during period of incubation, through febrile and eruptive stages, till all skin has fallen—twenty-one to twenty-eight days.

German measles—the same.

Whooping-cough—during the whole period of attack, even before "whooping" is noted—six to eight weeks.

Mumps—during whole illness, till ten days after glands have fallen—not less than twenty-eight days.

Enteric fever—during whole illness, until restoration to health.

Diphtheria—during whole illness, till ten days after the bacillus has disappeared from throat or nasal discharges —four to six weeks.

Erysipelas—from beginning of "rose" appearance of skin, till ten days after peeling of skin has ceased—not less than four weeks.

Before a patient can be safely restored to his friends, his person and clothing must have undergone adequate disinfection, and before the room can be considered habitable it, and its contents, must also be suitably disinfected.

These questions become of considerable importance in relation to school-attendance. How soon, after exposure to an infectious disease, may a child who has not before had the disease, be permitted to return to school? It is to be deplored that definite opinions do not obtain generally on his point, and the legislature has done nothing to produce uniformity. In England, the Medical Officers of Schools Association has helped on a forward movement in this direction by the code which it published some years ago. If carried out, it would at least secure uniformity of action, and even in this respect its object would be praiseworthy. We heartily concur in its provisions, the substance of which answers the above query, and which are as follow :—

After thorough disinfection of the person and clothing of a child, he or she may be permitted to return to school: In diphtheria, after 12 days; measles, 16; German measles, 16; small-pox, 18; chicken-pox, 18; scarlet fever, 14; whooping-cough, 21; mumps, 24.

### Midwifery Papers.

### X.—HÆMORRHAGE: POST-PARTUM.

Post - partum Hæmorrhage. — The chief cause of hæmorrhage after the birth of the child and before the placenta has been expelled is the prevention of uterine contraction by partially adherent or retained placenta. Blood is lost with great rapidity, and the midwife should get the placenta away and the uterus contracted as soon as possible. To do this never on any account pull on the cord. By so doing you may (*a*) increase the risk of hæmorrhage by tearing the uterine tissue as you drag away the adherent placenta; or (*b*) you may cause the inversion of the uterus, *i.e.*, turning the uterus inside out, by drawing down the fundus with the placenta. If you cannot "express" the placenta in the usual way, carefully insert your right hand into the uterus (introducing your fingers gathered together in the shape of a cone first) and feel if the membranes are still attached to the uterine wall. If so, gently detach them, taking care not to tear down any of the soft uterine tissue and so increase the hæmorrhage. When you have succeeded in detaching the placenta, you should make firm pressure with your left hand, which should have been grasping the fundus the whole time, and "express" your right hand and the placenta together. When you have removed an adherent placenta, it should be very carefully examined to see that no broken pieces of membranes are left behind. Another cause of post-partum hæmorrhage is irregular uterine contraction. The uterus contracts into the shape of an hour-glass, the placenta being retained in the upper portion which is relaxed, the cord only passing through the tight constriction in the body of the uterus. This form of irregular uterine contraction may result from (1) careless pressure on the fundus or (2) by pulling on the cord; also (3) by the too early administration of ergot. In "hour-glass contraction" the midwife should follow the cord to its insertion inside the contracted part of the uterus, gently inserting the fingers of her right hand (cone-shaped) past the contraction and dilating it by degrees. If the membranes are attached to the uterine wall, gently and gradually separate them, and express the hand and placenta in the manner described above.

In some cases severe hæmorrhage comes on after the child and the placenta have been normally expelled. The chief cause is uterine inertia (failure of the muscular contractions of the uterus). This condition may be induced by (1) protracted labour in which the uterus has been tired out by its efforts; (2) precipitate labour, the uterus has been too rapidly emptied, and sufficient time has not been given for complete separation of the placenta; (3) over-distension of the uterine walls by (*a*) twins or triplets, (*b*) excess of liquor amnii, (*c*) repeated pregnancies; (4) pressure of full bladder or rectum; (5) uterine fibroids. If your patient has a large flabby uterus and the contractions are feeble, be on the look-out for hæmorrhage. The pulse is another danger-signal; if it reaches over 100 beats a minute anticipate and be prepared for hæmorrhage.

Lacerations of the cervix, vagina, or perinæum cause hæmorrhage. In either case send for a medical man, and while waiting his arrival give a hot douche, or apply hot fomentations.

The great treatment of hæmorrhage is to stimulate contraction of the uterus by firm compression of the fundus, occasionally kneading the uterus. Contractions are excited by inserting the hand into the uterus and moving the hand about, carefully removing any clots which may have collected. Another method is inserting the right hand along the posterior wall of the vagina and pressing firmly against the lower posterior wall of the uterus, the left hand externally grasping the fundus, making firm counter pressure. A hot antiseptic douche (temp. 120°); ergot, 1 to 2 drachms; local applications of ice (introducing pieces in the vagina); bandaging of lower limbs, have all been recommended in the treatment of hæmorrhage. As a last recourse, a hot (115°) intra-uterine douche may be tried; but this should only be given by the nurse when other remedies have failed. The patient should be kept very quiet in a recumbent position, the head resting lower than the other parts of the body to prevent fainting. She should be lightly covered, and plenty of fresh air must be admitted into the room. If there should be signs of syncope, the cautious use of brandy is recommended. A medical man should, of course, at once be sent for. In case of post-partum hæmorrhage the midwife should bear in mind that on no account must she relax her external hold of the fundus, for by this pressure she prevents the relaxation of the uterine walls, and so assists the natural contractions of the uterus and the closure of the uterine blood-vessels.

Hæmorrhage is one of the most—if not the most—alarming complication of labour: a great stream of blood rushes from the gaping mouths of the enlarged uterine blood-vessels, and the surrounding bed-clothes are saturated in a moment. It is just now that the patient's life depends on the coolness and skill of her nurse. A doctor may be miles away, and the nurse has to depend on her own resources. The patient must not be left for one moment till the uterus is firmly contracted and the bleeding has quite ceased. Where bleeding, however slight, has occurred, extra watchfulness and care is needed during the first few days or weeks after the confinement.

In normal cases of labour if care is taken during the second and third stages of labour to ensure uterine contractions, and to express the placenta in due time with its membranes intact, the risk of hæmorrhage is not great. The binder should not be applied till half an hour after the birth, and the uterus should then be contracted into a hard ball the size of the fœtal head.

### Nurses in 1896—Their Quarters, Hours, and Food.

[These articles exhibit the actual condition of affairs in the spring of the present year.]

### HOSPITAL FOR SICK CHILDREN, GREAT ORMOND STREET.

### I.—Terms of Training.

Probationers are received for a term of three years' training at the Hospital for Sick Children, Great Ormond Street; candidates must be not less than 21 years of age nor more than 30. The preliminary month of probation may be extended by the matron at her discretion for a further period not exceeding one month. Candidates who are considered suitable are then recommended by her to the committee for appointment, and, if accepted, sign an agreement to remain in the service of the hospital for three years, dating from the day of appointment, the House Committee retaining the power to terminate the engagement without notice. Probationers can only break their engagement by special permission of the committee through the matron. At the end of the second year of service probationers may be appointed probationary staff nurse, if considered fit for the duties by the matron, but without extra pay or exemption from the lectures, classes, and examinations which probationers are expected to attend. Regular courses of lectures are given by members of the medical staff, and instruction is also given by the matron, home-sister, and the ward sisters in practical nursing, cookery, massage, &c. Examinations are held in June each year for the junior probationers, and every October there is an examination for nurses in their third year. Nurses who fail to obtain 35 per cent. of the available marks are considered to have failed, and are only allowed to present themselves for further examination by special permission. At the end of the three years' service nurses who have proved themselves efficient are entitled to certificates to the effect that they have "received three years' training in nursing medical and surgical cases, and are competent to undertake the nursing of sick children."

A certain number of paying probationers are admitted between the ages of 21 and 35, in the first instance for a term of three months, signing an agreement to abide by the rules of the hospital. If recommended by the matron, a paying probationer may be subsequently reappointed for a further term of nine months; at the end of the twelve months, if satisfactory, receiving a certificate from the matron to the effect that she has received one year's training in the nursing of sick children, but in no case is a certificate implying that she is a trained nurse given to a nurse who has not completed the full three years' curriculum. A paying probationer wishing to complete her training may, on the recommendation of the matron, at the end of the twelve months be transferred to the regular staff, ranking and receiving pay as a probationer in her second year. No difference is made with regard to the details of training and ward work between ordinary and paying probationers. The fee for paying probationers is a guinea a week, payable in advance by quarterly instalments.

### II.—Hours of Work and Times off Duty.

The hours of duty for the day staff of nurses and probationers are from seven a.m. to eight p.m., the daily time off duty varying between two, three, or four hours. Allowing for meal times, and taking two hours off duty as the minimum allowance, the average working day for nurses and probationers is one of rather under ten hours. Probationers and nurses have one half day and one whole day off duty each month, and three weeks' holiday in the year. They are allowed to sleep away from the hospital on their monthly holidays, which count from six p.m. the evening of one day to nine p.m. the following day for probationers; an hour later for staff nurses. Sisters are on duty from nine a.m. to ten p.m., with three hours off duty daily, and once a month leave from Saturday to Monday.

Night nurses are on duty from eight p.m. to eight a.m., and are free from nine to twelve every morning. Annual holidays are for staff nurses and probationers three weeks, and for sisters one month.

### III.—Meals.

Breakfast for the day nurses is at twenty minutes to seven, luncheon is from nine to half-past nine, dinners at half-past twelve and one, tea half-past four and five, and supper at nine. Breakfast is a meat meal; soup, coffee or cocoa, and bread and butter are provided for lunch; dinner consists of meat, two vegetables, and pudding. Tea is served in the dining-room. Sisters breakfast at eight, have lunch from twelve to one, dinner at half-past six. Tea is served in their own rooms, and coffee in the dining-room at half-past eight p.m.

Nurses' night meals are in no case permitted to be taken in the wards, but are prepared in the ward kitchen at definite hours arranged by the night sister.

### IV.—Salaries and Uniform.

Ordinary probationers, when accepted by the committee, are paid at the rate of £8 the first year, £12 the second, and £18 the third, receiving in addition 1s. 8d. per week for washing. Staff nurses receive £25 for the first year's service, £26 for the second, and £27 for the third and subsequent years, with a similar allowance for laundry. Sisters' salaries are £35 per annum, and 2s. 3d. is allowed for washing each week.

Probationers are provided by the hospital with indoor uniform, three washing dresses, six linen aprons, three caps, and six collars. Indoor uniform is provided for all the regular staff, paying probationers being expected to defray the cost of their uniform and washing.

### V.—Nurses' Quarters.

The accommodation for the nursing staff at this hospital leaves very much to be desired. There is no separate home, the nurses' rooms being in various parts of the hospital building. For the most part nurses sleep two in a room divided by a curtain, but in one large dormitory no less than eight nurses are lodged. Miss Smedley says that this public apartment is popular as a sleeping place among the nurses, but on every ground such crowding and lack of privacy for women whose lives are spent in hospital wards is very greatly to be deplored. The furniture generally seems insufficient; there are no wardrobes for the nurses' dresses, which hang together on pegs under curtains. Ward sisters have sitting-rooms, very small ones, in some cases bed-sitting-rooms, outside their wards. The nurses' sitting-room space is very limited, but they have a fairly comfortable dining-room, and the sisters also have a separate dining-room. One room containing three or four beds is set apart as a sick-room for nurses. The Children's Hospital is an instance, one of many, where the staff has increased in proportion to beds, but not the space available, and a proper home with adequate separate sleeping accommodation is most urgently needed.

### Where to Go.

The winter sale of the Working Ladies' Guild will be held at 24, Park Lane, W., on December 8th, 9th, and 10th. H.R.H. Princess Christian will open the sale at twelve o'clock on Tuesday, the 8th. Admission: First day, 2s. 6d.; other days, 1s.; twelve to six. Tea stall.

National Orthopædic Hospital, Great Portland Street.—A sale of work will be held at this hospital on Friday and Saturday, December 4th and 5th, in support of the Aid Fund, and for the supply of additional comforts for the patients. The sale will be open each day from two to six. Admission, 1s.; children, half-price. Contributions of clothing, flowers, eggs, &c., will be gratefully received by the matron.

### A Book and its Story.

### THE MURDER OF DELICIA.

It is unnecessary to waste many words in describing the actual plot of this novel,* for the story is common-place as common-place can be. The husband of Delicia, a guardsman, Wilfred de Tracy Gifford Carlyon by name, has married her for her money. After a time he tires of her, neglects her, and has a vulgar intrigue with La Marina, a dancer at the Empire. Delicia finds him out and refuses to pardon him. They separate, and she dies of a broken heart.

Such things have happened, are happening, and will happen to the end of time; and there is no excuse for the conduct of Carlyon, who is a selfish, cold-hearted man about town, of the ordinary type. But the authoress has made this story a peg on which to hang a number of statements and theories with which we utterly disagree. Anticipating these objections at the hands of her readers, the authoress remarks in her preface, "I have to say in reply that the two chief personages in my story, namely, Lord Carlyon and his wife, are drawn strictly from the life, and that both the originals have some years since departed from this scene of earthly contest and misunderstanding. The murder of Delicia was consummated at the hands of her husband precisely in the way I have depicted it. There are thousands of such 'murders' daily happening among us—murders which are not considered 'cruelty' in the eyes of the law. There are any number of women who work night and day with brain and hand to support useless and brainless husbands; women whose love never falters, whose patience never tires, and whose tenderness is often rewarded only by the most callous neglect and ingratitude. I do not speak of the countless cases among the hard-working millions, whom we elect to call the 'lower classes,' where the wife, working from six in the morning till ten at night, has to see her hard earnings snatched from her by her 'better' half and spent at the public-house in strong drink, despite the fact that there is no food at home, and that innocent little children are starving. These instances are so frequent that they have almost ceased to awaken our interest, much less our sympathy. In my story I allude principally to the 'upper' ranks, where the lazy noodle of an aristocrat spends his time, first, in accumulating debts, and then in looking about for a woman with money to pay them—a woman upon whose income he can afterwards live comfortably for the rest of his worthless life." Miss Corelli has deserved, and achieved, an immense success as a writer of fiction. This is not to be wondered at, for she has imagination, a rare gift of style, and, we fancy, immense industry. The result of this combination of qualities is that her books are bought up as soon as they are published, and she is probably, at the present moment, more popular than any other living novelist. But what is the result? Instead of being gratified at her own popularity, and acknowledging that talent is appreciated whether it is possessed by a woman or a man, she never tires of proclaiming that men—the critics, we presume, in particular—are burning with jealousy and are leagued together for the purpose of abusing and running down any woman who takes pen in hand. "If a woman does anything out of the common in the way of art or literature she is immediately judged by men as being probably without tenderness, without permanence in her work, and certainly without personal beauty." From that text we had a pretty long sermon preached in "The Sorrows of Satan," and we venture to say that nothing more inconsistent with fact was ever written.

Is there a man in England who does not acknowledge the genius of Jane Austin, Charlotte Brontë, George Eliot, and of others, whose names are too numerous to mention? Or, in a word, which, of all the critics, have not dealt as fairly with the women as with the men? It is a departure from truth to say that when a woman writes men say or think she is "unsexed," to use Miss Corelli's own word. In every department of art and literature the work of women is welcomed, and, as no one knows better than Miss Corelli herself, paid for—a very practical test—on the same scale as work of a similar quality when produced by men.

But the "Murder of Delicia" is not meant to teach the lesson that men are cruel and base to the woman who writes so much as the other lesson that men are, as a rule, mercenary fortune-hunters.

"To put it bluntly," says Miss Corelli, "a great majority of the men of the present day want women to keep them"—not some men, or even many men, be it observed, but most men! What a perversion of the truth is this! and the context is equally absurd. "In cases where the woman's intellectual ability is brought into play, and where the financial results of her brain work are such that they enable her husband to live as he likes, he will frequently express regret, in grandiloquent terms, that there should be any women who 'want to be clever'; they are always 'unsexed.' This word 'unsexed' is always cast at brilliant women by every little halfpenny ragamuffin of the press that can get a newspaper in which to hide himself for the convenience of throwing stones. The woman who paints a great picture is 'unsexed'; in fact, whatever woman does that is higher and more ambitious than the mere act of flinging herself down at the feet of man, and allowing him to walk over her, makes her, in man's opinion, unworthy of his consideration as woman; and he fits the appellation 'unsexed' to her with an easy callousness which is as unmanly as it is despicable."

We have quoted this passage because, taken in conjunction with the other quotation about the "majority" of men, it contains the gist of Miss Corelli's position, which comes to this—that most men wish to live on the earnings of their womenfolk, and when they can do so they turn round and bite the hand that feeds them. "It is not a manly or noble desire; but as the kind of men I mean have neither the courage nor the intelligence to fight the world for themselves, it is, I suppose, natural for such inefficient weaklings that they should—seeing the fierce heat and contest of competition in every branch of modern labour—gladly sneak behind a woman's petticoats to escape the general fray." In the first place, for every man who lives on the earnings of a woman, there are, at least, a thousand women who live on the earnings of their husbands; and in the second place, we do not agree with the statement, that in the case of our modern women writers, whose brains bring in means to let their husbands live "surrounded with every ease and comfort," it is the custom of these fortunate gentlemen to go to "clubs," and sneer at their wives. The common-sense of the reading public revolts against such monstrous absurdities; and in making these wild statements Miss Corelli is merely "going one better" than Don Quixote. That old hero first sets up his windmill, and then proceeds to attack it. The only effect which these wholesale accusations have is to set people wondering what can be the special grievance against mankind which has stung the writer to the quick, and driven her to dip her pen in gall. The story of Delicia is tragic beyond words; but it would have been more powerful if it had been allowed to teach its own sad lesson without exaggerated comments. After all, in fiction, as in oratory, the highest art is to conceal art.

* "The Murder of Delicia." Marie Corelli. (London: Skeffington and Sons, 1896.)

### Wants and Workers.

Parish Nurse, Castle Morton, Malvern, will be very glad of any old clothing, especially boots and shoes, for the most destitute amongst her patients. The parish is a very poor one.

### Everybody's Opinion.

[Correspondence on all subjects is invited, but we cannot in any way be responsible for the opinions expressed by our correspondents. No communication can be entertained if the name and address of the correspondent is not given, or unless one side of the paper only is written on.]

### ORPHANS CAGED.

We had not intended to allow this correspondence to go any further. The medical officer of St. Mary's Home, Broadstairs, has, however, gone to the trouble of providing us with a photo-block of two of the "cubicles," with which we will deal next week.—Ed. *T. H.*

### THE NURSES AND QUEEN VICTORIA.

"A Pension Fund Nurse" writes: It seems to me that a shilling a year is a very small amount to fix as the minimum nurses' subscription towards helping the benevolent fund branch in commemoration of the Queen's long reign. Might it not be just doubled? Surely if a nurse can spare one shilling she might spare two, and the result would seem more worth the effort.

### THE PREVENTION OF INSANITY.

Miss Howarth writes: It has occurred to me that you would perhaps be able and willing to help me in a matter which I have much at heart. I will try and explain it as briefly as possible. Since I took up work among the insane, about eight years ago, I have been very much impressed with two facts. First, how much insanity is preventable; and, secondly, how profoundly ignorant most people are about insanity, its usual causes, and the ways in which it might be prevented. It is not only those who nurse the insane who require instruction, it is the general public, the people who themselves inherit insane tendencies, who have eccentric and hysterical relatives, and who have the care and training of children who are epileptic, or hysterical, or highly nervous. My own experience is that such people and such children are always treated in exactly the wrong way, so as to increase instead of diminishing their liability to become insane. Now it seems to me that this general public never reads such papers as the *Lancet* or The Hospital, and never has the opportunity of instruction on this very important subject. Would it not be possible to publish a collection of articles or short essays in a cheap form, written without technicalities, so that everyone might both understand and be interested, and so arouse some at least to a sense of personal responsibility, to make people feel that each individual can do something to prevent the increase of insanity? The writing of such a work would not be very difficult, I should think; it might be done by more than one person, each contributing one or more articles. The difficulty would be, I suppose, in the publishing and scattering broadcast, as to which I myself have, unfortunately, no facilities or opportunities. I find it not easy, mereover, to get anyone to help me, yet, as I said before, I am very much bent on doing something towards the attainment of my object, and I do not think I shall ever cease to try.

### Presentations.

Miss A. E. Coke, who has lately resigned her position of matron at the Windsor Royal Infirmary, after holding it for ten years, was presented before she left with an illuminated address, expressive of appreciation of her past services and the efficiency of the superintendence, accompanied by a handsome dressing bag, and a cheque for £10. The presentation was made in the board-room of the hospital. The Dean of Windsor, the Vicar, the Rev. J. H. Ellison, Canon Gee, and Dr. W. Fairbank were present amongst others. Many cordial good wishes for Miss Coke's future success were expressed, and regret at her departure from Windsor.

### Private Nursing in Australia.

Nursing in Australia is not a bed of roses—indeed, sometimes not a bed at all. We know some people on this side of the world who do not know the difference between a mere mortal woman and a steam engine, but the case which happened to an English nurse in Sydney, when she nursed a typhoid patient from Sunday morning till Tuesday mid-day without a break, is, we hope, unparalleled. This lady, who has eleven years' experience of Australian nursing, writes to us giving some details of the state of things in New South Wales. In the first place, the supply is greater than the demand. This puts the nurse at the mercy of the unscrupulous, who haggle with her to abate her fee from the two guineas a week, which is the nominal charge. Nurses with private means or influence may stand against this sweating, but most of them must take what they can get. Even so, there is not work enough for all, and as a result nurses may be seen going about from door to door selling things on commission or acting as agents for various trumpery inventions. They wear their uniform while thus engaged, which will surely be the means of making self-respecting nurses give up the wearing of a special or distinctive costume.

Yet our correspondent thinks there is work to be done and a living to be made by nurses who have courage and energy enough to face life in the bush townships. Private nursing in our acceptation of the word, would be unendurable to a refined woman, for even people who are not really poor live miserably—parents and a large family dwelling in an earthen-floored hut of one, or at most two rooms. But if a nurse, or perhaps two, took a lodging for themselves and went in for district nursing, not necessarily gratuitous, they might do good work, and, perhaps, develop a higher ideal of comfort and refinement among their patients' families. But it is well that it should be known that nursing in the colonies is not such a pleasant or profitable business as is generally supposed.

### St. Bartholomew's Nurses in Danger.

A rather serious fire broke out early on Wednesday morning at the houses in Smithfield occupied by nurses attached to St. Bartholomew's Hospital. It is reported that some of the nurses were rescued with difficulty, great credit being due to the porters, who made plucky and successful efforts to bring them out of danger, at considerable risk. One of the houses was almost gutted, two floors being burnt out, and the roof partially destroyed.

### Minor Appointments.

Dewsbury and District General Infirmary.—Miss Sarah Cash has been appointed Charge Nurse at this infirmary. She received her training at the Royal Infirmary, Halifax.

Blaby Isolation Hospital, Leicester.—Nurse E. A. Boutel has been appointed Nurse-in-Charge of this isolation hospital. She was trained at the Lambeth Infirmary, where she worked for three years as probationer and staff nurse. Recently Nurse Boutel has been working for the Institution of Trained Nurses at Leicester.

Chelsea Infirmary.—Miss Eleanor Barton has been appointed Superintendent of the new Nurses' Home at this Infirmary. She was trained at St. Bartholomew's Hospital, and afterwards held the position of staff nurse at the Royal Hants Infirmary, Winchester. Miss Barton also took a first-class in the examination for women at Dublin University, and has had useful experience as private secretary to her uncle, the Archbishop of Dublin.

### For Reading to the Sick.

### THE SHADOW OF THE FUTURE.

**Verses.**

My soul, there is a country
Afar beyond the stars,
Where stands a winged sentry
All skilful in the wars:

There above noise and danger,
Sweet Peace sits crowned with smiles,
And One born in the manger
Commands the beauteous files.

He is thy gracious friend,
And—O my soul, awake!—
Did in pure love descend
And die here for thy sake.

If thou can'st get but thither,
There grows the flower of Peace,
The rose that cannot wither,
Thy fortress and thy ease.

Leave then thy foolish ranges,
For none can thee secure
But One, who never changes,
Thy God, thy life, thy cure.

—*H. Vaughan*, 1650.

Lord, what a change within us one short hour
Spent in Thy presence will prevail to make,
What heavy burdens from our bosoms take,
What parched grounds refresh as with a shower;
We kneel, and all around us seems to lower,
We rise, and all the distance and the rear
Stands forth in sunny outline brave and clear;
We kneel how weak, we rise how full of power.
Why should we do ourselves this wrong
Or others?—that we are not always strong,
That we are ever overborne with care,
That we should ever weak or heartless be,
Anxious or troubled, when with us is prayer,
And joy, and strength, and courage are with Thee.

—*H. C. Trench.*

**Reading.**

He knows what is in the darkness."—*Dan. ii. 22.*

When our Lord steadfastly set his face to go to Jerusalem He knew what was at the journey's end. Does not the story of His life, from this point onwards, go home to those who carry about with them some secret dread of approaching suffering, and who need His special help to enable them to face the intermediate routine of daily life, and to preserve a spirit of "cheerful yesterdays and confident to-morrows?" Very often a hidden burden of this kind is borne in silence for the sake of others. Very often you carry a burden of this sort (and many a smaller one also, such as a headache) in silence for unselfish reason; and yet you unreasonably expect from those around that tenderness and sympathy which they would be eager to give had they been confided in; but, as it is, they know of no reason for bearing with your moods. . . . Perhaps Jesus knows what it is like, when you, conscious of the dark cloud hanging over you, are tempted to wonder fretfully at the unfeelingness of those around you, who are absorbed in some trifle of sorrow or gladness which touches themselves. But there was no touch of preoccupation or of hurt feeling in our Lord's treatment of those who were so "stupid at the heart" as never to feel a shadow on His path.—*Miss L. E. M. Soulsby.*

### Novelties for Nurses.

### A NEW "BABY-HOLDER."

"Brooke's Baby-Holder" is, we understand, the invention of a nurse, and is an ingenious contrivance for carrying a baby securely, while taking its weight off the nurse's or mother's arms, and leaving their hands free. The holder, which is made of stout twill calico or similar material, and is easily washed, fastens round the waist, with shoulder straps adjustable keeping baby in position, in what might be described as a little hammock, fitted with a reversible wedge-shaped pillow, to support the spine and prevent the child's head falling back. The cushion prevents the straps tightening on the child. For carrying a baby out-of-doors, or walking about with a fractious child, the arrangement is excellent, the position of the little one being quite safe and comfortable, while adding to the convenience of its nurse. The holder may be obtained from Mr. F. Cresswell, 15, Thornhill Crescent, Caledonian Road, N.; price 5s. 6d.

### Appointments.

### MATRONS.

Lewes Dispensary and Infirmary and Victoria Hospital.—Miss Mary P. Wood has been chosen to fill the post of Matron at this institution. She was trained at the Sussex County Hospital, subsequently being appointed sister of the children's ward, and has since been engaged in private nursing.

### Notes and Queries.

The contents of the Editor's Letter-box have now reached such unwieldy proportions that it has become necessary to establish a hard and fast rule regarding Answers to Correspondents. In future, all questions requiring replies will continue to be answered in this column without any fee. If an answer is required by letter, a fee of half-a-crown must be enclosed with the note containing the enquiry. We are always pleased to help our numerous correspondents to the fullest extent, and we can trust them to sympathise in the overwhelming amount of writing which makes the new rules a necessity. Every communication must be accompanied by the writer's name and address, otherwise it will receive no attention.

*Doll Nurses.*

(47) Can you tell me where I can get a doll nurse's chatelaine?—*Nurse R.*

Ask at some large toy shop, such as Hamley's, in Regent Street.

*Hospital Floors.*

(48) Will some readers of The Hospital advise a matron how best to treat a new pitch pine block floor?—*Miss H.*

Perhaps someone will make suggestions. A thorough soaking with good varnish, carefully applied, and subsequent polishing with bee's wax and turpentine, has been recommended for deal floors. The varnish permeating the wood, prevents shrinkage, and a good smooth surface is obtained. The first effect may not be very nice to look at, but time and polishing will improve that.

*Queen's Nurses.*

(49) Will you let me know what are the duties of Jubilee Nurses, and to whom should I apply for a post in England or Ireland? I am a private nurse.

You had better write for information to the Secretary, Queen Victoria' Jubilee Institute for Nurses, St. Katharine's Royal Hospital, Regent's Park, N.W. Six months' approved training in district work is an essential qualification, in addition to at least one year's general training, and nurses in country districts must have three months' midwifery training as well.

*District Nursing.*

(50) Can you give me the name of an infirmary which has a district nurse residing in it?—*M. E. C.*

We have heard that this plan is followed at the Halifax Infirmary. Perhaps some of our readers may be able to give other instances.

*Certificate or Testimonial.*

(51) A correspondent writes: I received my training in a provincial general hospital, the term of training being two years. Certificates are not given, only testimonials. I am told it is a great disadvantage not to have a certificate. Could I obtain one by attending lectures at any hospital or institution?

The value of a satisfactory testimonial from the matron of a provincial hospital of recognised standing would be everywhere recognised. You do not give the name of the hospital, so we cannot judge of the value of your training. You could only obtain a "certificate" by entering a hospital again as probationer and following the whole course for which it is given. Your testimonials, which you say are "excellent," should enable you to get good work quite as well as the possession of a printed form of certificate.

*Male Nurses.*

(52) Can you tell me of any hospital which trains male nurses, or give me any information on the subject.—*J. W. T.*

There is no training school for male nurses in England. A few men are taken for training at the Hospital for Epilepsy and Paralysis, Queen Square, Bloomsbury, W.C.

